# Hospitalization of HIV positive patients in a referral tertiary care hospital in Antananarivo Madagascar, 2010-2016: Trends, causes and outcome

**DOI:** 10.1371/journal.pone.0203437

**Published:** 2018-08-30

**Authors:** Mihaja Raberahona, Tiana Razafinambinintsoa, Volatiana Andriananja, Njaratiana Ravololomanana, Juliana Tongavelona, Rado Rakotomalala, Johary Andriamamonjisoa, Radonirina Lazasoa Andrianasolo, Rivonirina Andry Rakotoarivelo, Mamy Jean de Dieu Randria

**Affiliations:** 1 Infectious Diseases Unit, University Hospital Joseph Raseta Befelatanana, University Hospital of Antananarivo, Antananarivo, Madagascar; 2 Faculty of Medicine, University of Antananarivo, Antananarivo, Madagascar; 3 Infectious Diseases Unit, University Hospital of Tambohobe, Fianarantsoa, Madagascar; 4 University of Fianarantsoa, Fianarantsoa, Madagascar; Stellenbosch University, Desmond Tutu TB Centre, SOUTH AFRICA

## Abstract

**Background:**

During the last few years, significant efforts have been made to improve access to antiretroviral therapy which led to dramatic reduction in AIDS-related events and mortality in HIV positive patients at the global level. However, current data in Africa suggested modest impact of widespread antiretroviral therapy scale-up especially regarding HIV-related hospitalization. In this study, we aimed to describe causes of hospitalization and factors associated with AIDS-defining events and inpatient mortality.

**Materials and methods:**

A retrospective study was performed on medical records of HIV positive patients admitted for at least 24 hours in the Infectious Diseases Unit of the University Hospital Joseph Raseta Befelatanana Antananarivo. Cause of hospitalization was considered as the main diagnosis related to the symptoms at admission. Diagnostic criteria were based on criteria described in WHO guidelines. AIDS-defining events were defined as diseases corresponding to WHO stage 4 or category C of CDC classification.

**Results:**

From 2010 to 2016, 236 hospital admissions were included. AIDS-defining events were the most frequent cause of hospitalization (61.9%) with an increasing trend during the study period. Tuberculosis (28.4%), pneumocystis pneumonia (11.4%), cerebral toxoplasmosis (7.2%) and cryptococcosis (5.5%) were the most frequent AIDS-defining events. Tuberculosis was also the most frequent cause of overall hospitalization. In multivariate analysis, recent HIV diagnosis (aOR = 2.0, 95% CI: 1.0–3.9), CD4<200 cells/μl (aOR = 4.0, 95%CI: 1.9–8.1), persistent fever (aOR = 4.4, 95%CI: 2.1–9.0), duration of symptoms≥ 6 weeks (aOR = 2.6, 95%CI: 1.2–5.4) were associated with AIDS-defining events. Overall inpatient mortality was 19.5%. Age≥55 years (aOR = 4.9, 95%CI: 1.5–16.6), neurological signs (aOR = 3.2, 95%CI: 1.5–6.9) and AIDS-defining events (aOR = 2.9, 95%CI: 1.2–-7.2) were associated with inpatient mortality.

**Conclusions:**

AIDS-defining events were the most frequent cause of hospitalization during the study period. Factors associated with AIDS-defining events mostly reflected delay in HIV diagnosis. Factors associated with mortality were advanced age, neurological signs and AIDS-defining events.

## Background

Despite global decrease in AIDS-related death and improvement of access to antiretroviral therapy (ART), eastern and southern Africa remains the most affected region with an estimated 19 million people living with HIV and 960 000 new infections in 2015 [1]. In the sub-Saharan region, Madagascar remains an exception with low HIV infection prevalence of 0.2% among adults aged 15 to 49 in 2016 and a concentrated epidemic profile. According to UNAIDS estimation, 31 000 (25 000–39 000) people were living with HIV in 2016 with 4 300 new HIV infections [2]. Since 2005, a national free ART program was implemented in Madagascar. In Madagascar, the universal access to ART regardless of CD4 count as per WHO guidelines was already adopted [3, 4] which will help the country to achieve the UNAIDS 90 90 90 target by 2020 [5]. The cumulative number of patients in care at the national level was 630 in 2007 and increased to 2 279 as of February 2017 (data from “Direction de Lutte contre les IST/SIDA”, Ministry of Public Health, Madagascar). However, compared to the estimated number of people living with HIV, the number of patients enrolled in care is very low which probably related to a low HIV testing rate. Indeed, a large proportion of patients remained undiagnosed and are likely to be at high risk of late and advanced presentation at enrollment into care due to delay in HIV diagnosis [6]. These patients with advanced diseases also have higher rate of hospital admissions and higher short-term and mid-term mortality with excess cost of medical care [7–11]. The introduction of highly active ART decreased hospitalization rates among HIV positive patient especially in developed countries [8, 12, 13]. However, in Madagascar, little is known about hospitalization in HIV positive patients.

In this study, we aimed to describe causes of hospitalization and factors associated with AIDS-defining events (ADE) and inpatient mortality.

## Materials and methods

### Setting

This study was conducted in the Infectious Diseases Unit of the University Hospital Joseph Raseta Befelatanana, a tertiary care hospital located in Antananarivo, Madagascar. This unit is a national referral center for HIV positive patients with the largest patient cohort in the country. This hospital has approximately 350 beds and includes all medical specialties and an emergency and intensive care medicine department. The Infectious Diseases Unit of this hospital provides outpatient and inpatient care as well as ART for HIV positive patients coming from Antananarivo but also from other regions of Madagascar. In Madagascar, people living with HIV are followed-up by trained physician in selected centers which are mostly secondary or tertiary level healthcare. ART are also provided in these centers. In general, patients who cannot be managed in primary or secondary healthcare facility or who require specialist care or platform for advanced diagnostics are referred to tertiary level hospital. Current guidelines recommend once-daily single tablet combination of tenofovir, lamivudine and efavirenz for adult patient as first-line regimen. ART are initiated in all patient regardless of CD4 count. Routine hematological and biochemical laboratory tests were available in this healthcare facility as well as serological tests for the diagnosis of viral hepatitis B, C and syphilis and conventional bacteriological examination, including blood culture. Direct microscopy for acid-fast bacilli in sputum and gastric aspirate was available but was not available for other types of samples in extrapulmonary tuberculosis. Furthermore, access to culture for the detection of *Mycobacterium tuberculosis* is limited. Direct microscopy for the detection of Pneumocystis jirovecii in induced sputum was available. Cryptococcal antigen test by lateral flow assay in serum and cerebrospinal fluid samples was also available in addition to India ink staining and fungal culture of cerebrospinal fluid. A point-of-care test (Alere Pima^TM^ CD4, Alere Inc., USA) was used to assess CD4 count. Polymerase chain reaction test as well as HIV viral load were not routinely available during the study period. Standard radiography, computed tomography and magnetic resonance imaging were available but their access is limited by their cost.

### Study design

We conducted a retrospective study from January 2010 to December 2016. Medical records of patients hospitalized more than 24 hours were included. We excluded medical records that lacked essential data for the purpose of the study, missing medical records and medical records of patients discharged against medical advice as they did not contain discharge diagnosis. Demographic characteristics, clinical data including symptoms on admission, duration of symptoms before admission, history of HIV and ART, WHO stage at entry, CD4 nadir, CD4 count on admission, diagnosis and WHO stage at discharge and outcome were extracted. We reviewed diagnosis at discharge from each medical record. We considered as cause of hospitalization the diagnosis which is consistent with the symptoms that justified hospital admission. When several diagnoses were identified during the same hospitalization, we considered as cause of hospitalization a disease according to an order priority rule described elsewhere: (1) WHO stage 4 opportunistic disease, (2) WHO stage 3 opportunistic disease, (3) other infection, (4) other cancer, (5) WHO stage 4 HIV-related wasting syndrome or cachexia, (6) Other disease, (7) non-specific WHO stage 3 events (persistent fever or weight loss > 10% or chronic diarrhea) [14]. The diagnosis can be confirmed or presumptive. Diagnostic criteria were based on presumptive and definitive criteria for HIV-related clinical events described in WHO guidelines [15]. ADE was considered as disease corresponding to WHO stage 4 or category C of CDC classification [15, 16]. Due to the retrospective design of the study, determining the causes of death were not always possible. Therefore, for the purpose of the study, we considered death associated with each cause of hospitalization. Based on previously described time definition, patients were considered as loss to follow-up before hospitalization when their last follow-up visit was ≥ 180 days before hospital admission [17]. ART discontinuation before hospitalization were considered as interruption of ART for more than one month before hospital admission. We considered as ART naïve, patients who had never initiated ART after HIV diagnosis.

### Data collection

Data was collected in an anonymized case report form from medical records for each hospitalization. A unique identifier was used for each patient. Authors involved in data collection had access to identifying information. The dataset used in this study were entirely de-identified to ensure confidentiality. All the authors were involved in the management and the follow-up of the patients of this study at one time or another and were trained for good clinical practice.

### Statistical analysis

Percentage and frequencies were used to describe categorical variables. Continuous variables were describes by median and interquartile ranges (IQR). Chi-squared test and Fischer’s exact test when appropriate were used to compare categorical variables. Wilcoxon-Mann-Whitney test was used to compare continuous variables. Trends over time periods were tested using chi-square test with linear-by-linear association for categorical variables. Factors associated with ADE and factors associated with in-hospital mortality were assessed. Variables were identified in univariate analysis. Variables identified with P < 0.1 in univariate analysis were entered into a logistic regression model using forward-stepwise selection (likelihood ratio) method. Variables with P < 0.05 were kept in the final model. Goodness-of-fit of the final model was confirmed by Hosmer-Lemeshow test. P < 0.05 was considered as significant. All statistics are two-sided. Statistical analysis was performed using SPSS 23.0 (IBM Corp., Armonk, NY).

### Ethics statements

All data were extracted from medical records and were based on routinely collected information. Collected data were anonymized to ensure patient confidentiality. This study does not involve supplementary intervention. Individual consent was not obtained. The National Ethic Committee (Comité d'Ethique de la Recherche Biomédicale auprès du Ministère de la Santé Publique) waived ethical approval application for this study due to its retrospective design as stated in letter N°75 MNSANP/CERBM.

## Results

Between January 2010 and December 2016, 252 hospital admissions of HIV positive patients among 6 187 total admission were recorded. Seven hospital admissions of HIV positive patients out of 986 (0.7%, 95% CI: 0.3–1.4) were recorded in 2010, 13 out of 1043 (1.2%, 95% CI: 0.7–2.0) in 2011, 13 out of 1050 (1.2%, 95% CI: 0.7–2.1) in 2012, 44 out of 885 (5.0, 95% CI: 3.7–6.6) in 2013, 47 out of 881 (5.3, 95% CI: 4.0–7.0) in 2014, 55 out 691 (8.0%, 95% CI: 6.1–10.2) in 2015 and 73 out of 651 (11.2%, 95% CI: 9.0–13.8) in 2016. Hospital admission of HIV positive patients accounted for 4.1% of overall hospital admission during the study period. Hospital admissions of HIV positive patients increased significantly from 0.7% in 2010 to 11.2% in 2016 (p < 0.001 for trend).

We included 236 hospital admissions corresponding to 178 patients for the analysis. Sixteen hospital admissions were excluded due to a discharge against medical advice (n = 11) and missing medical records (n = 5). Among the 178 patients, 137 patients had 1 hospital admission, 31 patients had 2 hospital admissions, 5 patients had 3 hospital admissions, 3 patients had 4 hospital admissions and 2 patients had 5 hospital admissions during the study period.

The characteristics of the patients at admission were described in Table 1. The diagnosis of HIV was made during hospital admission in 93 out of 236 hospital admissions (39.4%) and this proportion did not vary significantly over the study period (p = 0.743 for trend). When considering the first hospital admission during the study period, HIV infection was known before admission in 85 out of 178 hospital admissions (47.8%). Median time between HIV diagnosis and first hospital admission was 19 months (IQR: 0–47). Among these 85 hospital admissions, patients were lost of follow-up before admission in 33 out of 85 hospital admissions (38.8%) and were not on ART in 63 out of 85 admissions (74.1%) including ART discontinuation in 16 out of 85 admissions (18.8%) and ART naïve in 47 out of 85 admissions (55.3%). Patients with previously known HIV infection were on ART during the first hospital admission in 22 out of 85 hospital admissions (25.9%). Median duration between ART initiation and first hospital admission among these patients was 3.5 months (0–42).

**Table 1:** Patient characteristics at admission.

Characteristics	n (%)
Age in years (median, IQR)	37 (30–45)
Male	150 (63.6)
Residency (region)	
• Antananarivo	163 (69.1)
• Outside Antananarivo	73 (30.9)
Diagnostic of HIV before hospitalization	143 (60.6)
Delay between HIV diagnosis and hospitalization in months (median, IQR)	14 (2–49)
Delay between HIV diagnosis and hospitalization	
• < 1 month	24 (16.8)
• 1–6 months	33 (23.1)
• ≥ 6 months	86 (60.1)
ART before admission	68/236 (28.8)
Duration of ART before hospitalization in months (median, IQR)	3 (1–9.75)
Loss to follow-up before hospitalization	37/236 (15.7)
Discontinuation of ART before hospitalization	22/236 (9.3)
Not on ART on admission among patients with diagnostic of HIV before admission	75/143 (52.4)
Diagnostic of HIV during hospitalization	93/236 (39.4)
WHO stage at entry	
• Stage 1	12 (5.1)
• Stage 2	13 (5.5)
• Stage 3	77 (32.6)
• Stage 4	134 (56.8)
WHO stage at discharge	
• Stage 1	8 (3.4)
• Stage 2	11 (4.7)
• Stage 3	50 (21.2)
• Stage 4	167 (70.8)
CD4 count on admission (median, IQR) (n = 200)	119 (58–222.75)
• < 50 cells/μl	44 (22)
• 50–99 cells/μl	41 (20.5)
• 100–199 cells/μl	55 (27.5)
• 200–349 cells/μl	36 (18)
• 349–499 cells/μl	16 (8)
• ≥ 500 cells/μl	8 (4)
Length of hospitalization in days (median, IQR)	18 (9–29)
Duration of hospitalization	
• < 1 week	41 (17.4)
• 1–2 weeks	50 (21.2)
• 2–3 weeks	43 (18.4)
• 3–4 weeks	37 (15.7)
• ≥ 4 weeks	65 (27.5)
In-hospital death	46 (19.5)

This table describe characteristics at admission during 236 hospital admissions by 178 patients

Clinical findings at admission were fever in 167 hospital admissions (70.8%), weight loss in 107 (45.3%), impaired general condition in 139 (58.9%), neurological signs including headache, altered consciousness, seizures, focal neurological signs and behavioral disorders in 66 (28%), respiratory signs including cough, dyspnea, hemoptysis and chest pain in 108 (45.8%), digestive signs including diarrhea, abdominal pain and vomiting in 85 (36%) and dermatological signs including in 26 (11%). Median duration of symptoms before hospital admission was 3 weeks (IQR: 1–8). Median duration of symptoms was longer in hospitalization caused by ADE compared to non-ADE (4 weeks, IQR: 2–12 vs 2 weeks, IQR: 1–4; p < 0.001).

The proportion of patients with CD4 < 200 cells/μl on admission did not vary over time (p = 0.150 for trend). However, the proportion of patients with CD4 < 100 cells/μl and CD4 < 50 cells/μl on admission increased respectively to reach 57.9% (p = 0.006 for trend) and 34.2% (p = 0.039 for trend) in 2016.

Causes of hospital admissions were described in Table 2 and Table 3. ADE represented 146 out of 236 (61.9%) hospital admissions. From 2010 to 2016, hospital admissions for ADE increased from 42.9% to 71% (p = 0.008 for trend). Tuberculosis was the most frequent cause of hospital admission (28.4%). Hospital admissions due to tuberculosis remained stable over the study period (p = 0.840 for trend). However hospital admissions due to pulmonary tuberculosis decreased significantly from 28.6% to 1.4% from 2010 to 2016 (p = 0.025 for trend) and hospital admissions due to extrapulmonary tuberculosis increased from 14.3% to 23.2% during the study period but did not reach statistical significance (p = 0.066 for trend). Hospital admissions due to pneumocystis pneumonia (p = 0.232 for trend) and cerebral toxoplasmosis (p = 0.111 for trend) did not change over the study period. Hospital admissions due to cryptococcosis significantly increased to reach 8.7% of hospital admissions in 2016 (p = 0.029 for trend). During the study period, diagnosis of ADE was presumptive in 47.9% and confirmed in 52.1%. This proportion did not change significantly over the study period (p = 0.317 for trend). Among leading causes of ADE, diagnosis was confirmed in 18.5% (n = 5) of pneumocystis pneumonia, 52.4% (n = 11) of pulmonary tuberculosis, 47.8% (n = 22) of extrapulmonary tuberculosis, 70.6% (n = 12) of cerebral toxoplasmosis and 100% (n = 13) of cryptococcosis. Among non-ADE hospital admissions, proportion of non-AIDS-defining infections did not change over the study period (p = 0.160 for trend) and represented 9.3% of overall hospital admissions. Hospital admissions related to adverse drug reactions including those related to ART and to other drugs represented 6.8% of total admission. This proportion did not change significantly over the study period (p = 0.813 for trend). Median duration of hospitalization was longer for hospital admission related to ADE comparing to non-ADE (22 days, IQR: 14–33 vs 11 days, IQR: 6–20; p < 0.001).

**Table 2:** Trends in causes of hospital admissions related to AIDS-defining events.

	Overall	2010	2011	2012	2013	2014	2015	2016
	n (%)	n (%)	n (%)	n (%)	n (%)	n (%)	n (%)	n (%)
AIDS-defining events	146 (61.9)	3 (42.9)	4 (33.3)	5 (45.5)	25 (62.5)	26 (60.5)	34 (63)	49 (71)
• Tuberculosis	67 (28.4)	3 (42.9)	-	2 (18.2)	13 (32.5)	16 (37.2)	16 (29.6)	17 (24.7)
– Pulmonary tuberculosis	21 (8.9)	2 (28.6)	-	2 (18.2)	6 (15.0)	4 (9.3)	6 (11.1)	1 (1.4)
– Extrapulmonary tuberculosis	46 (19.5)	1 (14.3)	-	-	7 (17.5)	12 (27.9)	10 (18.5)	16 (23.2)
– Disseminated tuberculosis	17 (7.2)	1 (14.3)	-	-	2 (5.0)	4 (9.3)	3 (5.6)	7 (10.1)
– Nodal tuberculosis	7 (3)	-	-	-	2 (5.0)	3 (7.0)	1 (1.9)	1 (1.4)
– Pleural tuberculosis	7 (3)	-	-	-	1 (2.5)	2 (4.7)	2 (3.7)	2 (2.9)
– Tuberculous meningitis	6 (2.5)	-	-	-	1 (2.5)	-	3 (5.6)	2 (2.9)
– Miliary tuberculosis	6 (2.5)	-	-	-	1 (2.5)	1 (2.3)	1 (1.9)	3 (4.3)
– Peritoneal tuberculosis	1 (0.4)	-	-	-	-	1 (2.3)	-	-
– Urogenital tuberculosis	1 (0.4)	-	-	-	-	1 (2.3)	-	-
– Tuberculous spondylitis	1 (0.4)	-	-	-	-	-	-	1 (1.4)
• Pneumocystis pneumonia	27 (11.4)	-	2 (16.7)	-	5 (12.5)	4 (9.3)	4 (7.4)	12 (17.4)
• Cerebral toxoplasmosis	17 (7.2)	-	1 (8.3)	2 (18.2)	1 (2.5)	1 (2.3)	1 (1.9)	11 (15.9)
• Cryptococcosis	13 (5.5)	-	-	-	1 (2.5)	1 (2.3)	5 (9.3)	6 (8.7)
• Kaposi sarcoma	4 (1.7)	-	1 (8.3)	-	2 (5)	-	-	1 (1.4)
• HIV encephalopathy	3 (1.3)	-	-	-	-	-	3 (5.6)	-
• HIV-associated nephropathy	3 (1.3)	-	-	-	-	1 (2.3)	2 (3.7)	-
• Esophageal candidiasis	2 (0.8)	-	-	-	1 (2.5)	1 (2.3)	-	-
• Disseminated nontuberculous mycobacterial infection	2 (0.8)	-	-	-	-	1 (2.3)	1 (1.9)	-
• Cytomegalovirus retinitis	2 (0.8)	-	-	1 (9.1)	1 (2.5)	-	-	-
• Invasive non-typhoidal salmonella disease	2 (0.8)	-	-	-	-	-	2 (3.7)	-
• HIV-associated cardiomyopathy	1 (0.4)	-	-	-	-	1 (2.3)	-	-
• Non-Hodgkin lymphoma	1 (0.4)	-	-	-	-	-	-	1 (1.4)
• Bacterial meningitis	1 (0.4)	-	-	-	1 (2.5)	-	-	-
• HIV wasting syndrome	1 (0.4)	-	-	-	-	-	-	1 (1.4)

**Table 3:** Trends in causes of hospital admission related to non-AIDS defining events.

	Overall	2010	2011	2012	2013	2014	2015	2016
	n (%)	n (%)	n (%)	n (%)	n (%)	n (%)	n (%)	n (%)
Non-ADE	90 (38.1)	4 (57.1)	8 (66.7)	6 (54.5)	15 (37.5)	17 (39.5)	20 (37)	20 (29)
• Non-AIDS-defining infections	22 (9.3)	1 (14.3)	1 (8.3)	2 (18.2)	4 (10)	6 (14)	5 (9.3)	3 (4.3)
• Non-specific WHO stage 3 events ^a^	15 (6.4)	-	3 (25)	1 (9.1)	4 (10)	2 (4.7)	4 (7.4)	1 (1.4)
• Adverse drug reactions	11 (4.7)	-	1 (8.3)	1 (9.1)	4 (10)	2 (4.7)	-	3 (4.3)
• Neurological diseases	9 (3.8)	-	-	1 (9.1)	2 (5)	5 (11.6)	-	1 (1.4)
• Gastrointestinal diseases	8 (3.4)	2 (28.6)	1 (8.3)	-	-	1 (2.3)	2 (3.7)	2 (2.9)
• Adverse drug reactions to ART	5 (2.1)	-	-	-	-	1 (2.3)	2 (3.7)	2 (2.9)
• ART initiation	5 (2.1)	-	-	-	1 (2.5)	-	2 (3.7)	2 (2.9)
• Surgical pathology	5 (2.1)	-	1 (8.3)	1 (9.1)	-	-	1 (1.9)	2 (2.9)
• Non-AIDS-defining neoplasia	2 (0.8)	1 (14.3)	1 (8.3)	-	-	-	-	-
• Psychiatric diseases	2 (0.8)	-	-	-	-	-	1 (1.9)	1 (1.4)
• Cardiac diseases	1 (0.4)	-	-	-	-	-	1 (1.9)	-
• Other ^b^	5 (2.1)	-	-	-	-	-	2 (3.7)	3 (4.3)

^a^ Non-specific WHO stage 3 events were defined as persistent fever or weight loss > 10% or chronic diarrhea

^b^ including one case of immune reconstitution inflammatory syndrome

Table 4 described factors associated with ADE. In multivariate analysis, factors associated with ADE were delay between HIV diagnosis and hospitalization < 1 month (aOR: 2.0, 95% CI: 1.0–3.9), presence of persistent fever (aOR: 4.4, 95% CI: 2.1–9.0), duration of symptoms ≥ 6 weeks (aOR: 2.6, 95% CI: 1.2–5.4) and CD4 < 200 cells/μl (aOR: 4.0, 95% CI: 1.9–8.1).

**Table 4:** Univariate and multivariate analysis of factors associated with AIDS defining events as cause of hospital admission.

Variables	Non-ADE n (%)	ADE n (%)	OR (95% CI)	*P-value*	aOR (95% CI)	*P-value*
Age ≥ 35 years	48 (53.3)	94 (64.4)	1.6 (0.9–2.7)	*0*.*092*		
Male	58 (64.4)	92 (63)	0.9 (0.5–1.6)	*0*.*824*		
Diagnostic of HIV during hospitalization	21 (23.3)	72 (49.3)	3.2 (1.8–5.7)	*<0*.*001*		
First hospitalization	55 (61.1)	123 (84.2)	3.4 (1.8–6.3)	*<0*.*001*		
Time elapsed between HIV diagnosis and hospitalization < 1 month ^a^	28 (31.1)	89 (61)	3.4 (1.8–6.3)	*<0*.*001*	2.0 (1.0–3.9)	*0*.*037*
Not on ART before admission	47 (52.2)	121 (82.9)	4.4 (2.4–8.0)	*<0*.*001*		
CD4 < 200 cells/μl	43 (54.4)	97 (80.2)	3.2 (1.7–6.0)	*<0*.*001*	4.0 (1.9–8.1)	*<0*.*001*
Persistent fever	46 (51.1)	121 (82.9)	4.6 (2.5–8.4)	*<0*.*001*	4.4 (2.1–9.0)	*<0*.*001*
Weight loss	28 (31.1)	79 (54.1)	2.6 (1.5–4.5)	*0*.*001*		
Impaired general condition	37 (41.1)	102 (69.9)	3.3 (1.9–5.7)	*<0*.*001*		
Duration of symptoms ≥ 6 weeks	17 (18.9)	55 (37.7)	2.6 (1.4–4.8)	*0*.*002*	2.6 (1.2–5.4)	*0*.*014*

R^2^ = 0.300, P-value = 0.330 (Hosmer-Lemeshow test). OR: odds-ratio, aOR: adjusted odds-ratio, ADE: AIDS defining events

^a^ including patients with diagnosis of HIV infection during hospital admission

In-hospital mortality occurred in 46 out of 236 hospital admissions (19.5%). In-hospital mortality significantly increased to reach 30.4% in 2016 (p = 0.006 for trend). Mortality rate was significantly lower in non-ADE hospital admissions compared to ADE hospital admissions (10% vs 25.3%, p = 0.004). Mortality rate was 22.4% in tuberculosis (19% in pulmonary tuberculosis and 23.9% in extrapulmonary tuberculosis), 25.9% in pneumocystis pneumonia, 29.4% in cerebral toxoplasmosis and 61.5% in cryptococcosis. In multivariate analysis, factors associated with mortality (Table 5) were age ≥ 55 years (aOR: 4.9, 95% CI: 1.5–16.6), presence of neurological signs (aOR: 3.2, 95% CI: 1.5–6.9) and diagnosis of ADE (aOR: 2.9, 95% CI: 1.2–7.2).

**Table 5:** Univariate and multivariate analysis of factors associated with in-hospital death.

Variables	Survived n (%)	Deceased n (%)	OR (95% CI)	*P-value*	aOR (95% CI)	*P-value*
Age ≥ 55 years ^a^	3 (4.7)	8 (17.4)	4.2 (1.5–11.7)	*0*.*003*	4.9 (1.5–16.6)	*0*.*010*
Male	124 (65.3)	26 (56.5)	0.7 (0.4–1.3)	*0*.*269*		
Neurological signs ^a^	45 (23.7)	21 (45.7)	2.7 (1.4–5.3)	*0*.*003*	3.2 (1.5–6.9)	*0*.*003*
Respiratory signs	85 (44.7)	23 (50)	1.2 (0.6–2.4)	*0*.*520*		
Digestive signs	67 (35.3)	18 (39.1)	1.2 (0.6–2.3)	*0*.*624*		
Weight loss ^a^	81 (42.6)	26 (56.5)	1.7 (0.9–3.4)	*0*.*090*		
Impaired general condition	108 (56.8)	31 (67.4)	1.6 (0.8–3.1)	*0*.*192*		
Duration of symptoms ≥ 6 weeks	56 (29.5)	16 (34.8)	1.3 (0.6–2.5)	*0*.*483*		
WHO stage 4 at discharge ^a^	127 (66.8)	40 (87)	3.3 (1.3–8.2)	*0*.*007*		
AIDS defining events ^a^	109 (57.4)	37 (80.4)	3.1 (1.4–6.7)	*0*.*004*	2.9 (1.2–7.2)	*0*.*021*
CD4 < 50 cells/μl ^a^	31 (19.5)	13 (34.5)	2.2 (1.0–4.8)	*0*.*043*		

R^2^ = 0.200, P-value = 0.609 (Hosmer-Lemeshow test). OR: odds-ratio, aOR: adjusted odds-ratio

^a^ variables included in multivariate analysis

## Discussion

This study highlighted a significant increase in HIV positive patient hospital admissions during the study period when considering the proportion of HIV positive patient hospital admissions compared to overall hospital admission as well as the absolute number of HIV positive patients admitted. However, we also noted a decrease in total admission during the study period which could be partly explained by the increase of hospital admission of HIV positive patients as they often required longer hospital stay, septic isolation and individual room for confidentiality purpose. Indeed, a previous study in high prevalence countries in Africa confirmed the impact of HIV on hospital bed occupancy [18].

ADE were the most common causes of hospital admission among HIV positive patients. Furthermore, tuberculosis was the leading cause of hospital admission among ADE but also when considering overall causes of hospital admission. Indeed, ADE and especially tuberculosis remained the most common causes of hospital admission worldwide and in Africa [14, 19, 20]. In Madagascar, incidence rate of tuberculosis was estimated to 237 (153–338) per 100 000 among general population of which 5.6 (2.5–9.8) per 100 000 occurred among HIV positive in 2016 [21]. A decrease in hospitalizations due to ADE has been observed since widespread of highly active ART use in middle and high-income countries [8, 13, 22, 23]. However, hospitalization rate and inpatient mortality remained high in Africa despite effective ART scale-up [24]. During the study period, median of CD4 on admission remained very low and proportion of patients admitted with CD4 < 100 cells/μl and < 50 cells/μl increased constantly which probably reflects an increasing trend of patients with severe immunosuppression condition, advanced stage of disease and late diagnosis especially among those with unknown HIV infection on admission. This situation also explains the increase in ADE hospital admissions. In addition, 39.4% of the patients were diagnosed as HIV positive patients during hospital admission which is slightly higher than reported in other African countries [19]. This situation may suggest gap in primary health care services as some patients received care in these settings before admission without being tested for HIV despite suggestive clinical signs. But in general, it also suggests a gap in provider-initiated testing and counseling for HIV. Moreover, it probably reflects lack of awareness of HIV among general population. In the Infectious Diseases Unit of the University Hospital Joseph Raseta Befelatanana, HIV testing is suggested to any inpatients at their admission, regardless of their clinical symptoms, their condition or whether they belong to at-risk group. This approach largely contributed to the increasing trend of HIV diagnosis in our unit. Such strategy could be suggested to other healthcare facility to improve HIV diagnosis. Indeed, routine HIV screening remains a cost-effective strategy even in a setting with HIV prevalence <0.1% [25]. Most of the patients were not on ART on admission which included patients who were diagnosed during hospital admission but also 52.4% of patients with known HIV infection on admission. Delay in ART initiation may contribute to the lack of ART initiation in patients with known HIV infection before admission. Indeed, during the study period, the majority of HIV positive patients were eligible to ART according to their CD4 count as recommended by the successive WHO guidelines. Madagascar has currently adopted “Treat all” policy according to WHO recommendation which is expected to decrease HIV-related morbidity and mortality. However, late presentation to care is common in African countries and may diminish the impact of this strategy [6, 26, 27]. Consequently, hospital admissions related to ADE may continue to increase in the coming years as suggested in our study. In addition, most of the patients previously on ART have not been treated for a long time as suggested by the low median duration of ART before admission. Moreover, on hospital admission, patients were generally young as suggested by median age. These facts may also explain the scarcity of diseases related to aging or metabolic and cardiovascular disorders that may be associated with long-term ART.

We identified factors associated with ADE as final diagnosis among HIV positive patients during hospital admission. As expected, patients with CD4 < 200 cells/μl were likely to have ADE. Others factors including recent diagnosis of HIV (time elapsed between HIV diagnosis and hospital admission < 1 month), presence of persistent fever at admission and duration of symptoms ≥ 6 months were also identified as predictive of ADE during hospital admission. In resource-limited settings with poor access to diagnostic confirmation, laboratory and imaging facilities, the presence of these features could be helpful in algorithmic approach for clinical management of the patients. Thereby, ADE related to initial symptoms of patients should be considered whenever these features are present. In addition, tuberculosis should be considered whenever clinical findings are consistent as tuberculosis may be difficult to diagnose. Indeed, only 49.3% of tuberculosis cases were confirmed in our study. According to the current WHO guidelines, ART is initiated regardless of CD4 count and monitoring CD4 count can be stopped in patients who are stable and virally suppressed [28]. However, we emphasize the importance of CD4 count in HIV positive inpatients as it gives valuable information on patient immune status and has major role in algorithmic approach for the diagnosis of ADE. Indeed, our study highlighted CD4 < 200 cells/μl is an independent factor associated with ADE.

The overall inpatient mortality was 19.5% in our study which was lower compared to what was reported in the African region where inpatient mortality was 31% [19]. However, we noted an increase trend during the study period which likely due to increase in ADE. In-hospital tuberculosis-related mortality was similar to other African countries [20, 29]. However, mortality related to cryptococcosis was very high and may be related to unavailability of amphotericin-based induction therapy in our setting. Older age, presence of neurological signs and ADE were identified as independently associated with in-hospital death. These factors were also reported in other studies [14, 24, 30]. Presence of neurological signs at admission was associated with higher mortality which is likely due to high mortality rate in central nervous system ADE such as cryptococcosis and cerebral toxoplasmosis. In our setting, patients with neurological signs were at high risk of diagnostic delay due to unavailability or poor access to cerebral imaging which may contribute to increase mortality.

The capacity of the referral centers for the management of HIV positive patients should be strengthened due to the upward trend of ADE-related hospital admissions which reflects diagnosis of HIV infection at an advanced stage. Provider-initiated HIV testing and counseling should be improved in as it substantially improves screening rate. However, Madagascar should intensely improve primary health care and community HIV testing to reduce the burden late and advanced presentation of HIV that contribute to increase hospital admission of HIV positive patients.

Our study had several limitations due to its retrospective design. Some opportunistic infections such as CMV-associated meningitis and pneumonia or progressive multifocal leukoencephalopathy could not be diagnosed as tests for confirmation were not available. Disseminated non-tuberculous mycobacteria infection could be underestimated due to poor access to culture confirmation. Clinical features related to immune reconstitution syndrome were also difficult to assess which probably led to underestimate its burden. Otherwise, data incompleteness may have limited the assessment of some factors associated with ADE or in-hospital death. The small sample size may also have limited the estimation of disease-specific mortality rate. For the purpose of this study, we were also unable to assess the presence of several opportunistic infections in the same patients which may have an impact on the prognosis. Finally, the cause of death in our study was only based on retrospective review of medical records which may not take into account the exact underlying condition which lead to death.

## Conclusions

In this study, ADE were the main cause of hospitalization in HIV positive patient with an increase trend during the study period. Tuberculosis was the leading cause of ADE as well as overall cause of hospitalization. Recent HIV diagnosis, low CD4, persistent fever and long duration of symptoms before admission were associated with ADE as cause of hospitalization. Overall mortality was 19.5%. Mortality was associated with advanced age, presence of neurological signs and ADE.

## Supporting information

S1 Appendix De-identified raw dataset. (XLSX)
